# Germline genetic variation and predicting immune checkpoint inhibitor induced toxicity

**DOI:** 10.1038/s41525-022-00345-6

**Published:** 2022-12-24

**Authors:** Ik Shin Chin, Aman Khan, Anna Olsson-Brown, Sophie Papa, Gary Middleton, Claire Palles

**Affiliations:** 1 Institute of Cancer and Genomic Sciences, University of Birmingham, Birmingham, UK; 2 The Clatterbridge Cancer Centre, Wirral, UK; 3 The University of Liverpool, Liverpool, UK; 4 School of Cancer and Pharmaceutical Studies, King’s College London, London, UK; 5 Institute of Immunology and Immunotherapy, University of Birmingham, Edgbaston, Birmingham, UK; 6 Queen Elizabeth Hospital, Birmingham, UK

**Keywords:** Predictive markers, Cancer genomics, Cancer immunotherapy

## Abstract

Immune checkpoint inhibitor (ICI) therapy has revolutionised the treatment of various cancer types. ICIs reinstate T-cell function to elicit an anti-cancer immune response. The resulting immune response can however have off-target effects which manifest as autoimmune type serious immune-related adverse events (irAE) in ~10–55% of patients treated. It is currently challenging to predict both who will experience irAEs and to what severity. Identification of patients at high risk of serious irAE would revolutionise patient care. While the pathogenesis driving irAE development is still unclear, host genetic factors are proposed to be key determinants of these events. This review presents current evidence supporting the role of the host genome in determining risk of irAE. We summarise the spectrum and timing of irAEs following treatment with ICIs and describe currently reported germline genetic variation associated with expression of immuno-modulatory factors within the cancer immunity cycle, development of autoimmune disease and irAE occurrence. We propose that germline genetic determinants of host immune function and autoimmune diseases could also explain risk of irAE development. We also endorse genome-wide association studies of patients being treated with ICIs to identify genetic variants that can be used in polygenic risk scores to predict risk of irAE.

## Introduction

Immune checkpoint inhibitors (ICI) are monoclonal antibodies that release the brakes off immune checkpoints such as cytotoxic T lymphocyte antigen (CTLA-4), programmed cell death 1 (PD-1), programmed cell death ligand 1 (PD-L1) and lymphocyte-activation gene 3 (Lag-3). They prevent the immune escape of cancer cells and ultimately cause cancer cell death^1^. They have revolutionised the management of several cancer types including melanoma, lung and urological cancers, which have historically had dismal prognoses in the metastatic setting. Commonly used ICIs include anti-PD-1 (nivolumab, pembrolizumab), anti-PD-L1 (atezolizumab, durvalumab, avelumab) and anti-CTLA-4 (ipilimumab) agents. Despite their notable successes in improving patients’ survival, ICI-induced toxicities, also known as immune-related adverse events (irAEs), can be life-changing and in some cases fatal. As the indication for using ICIs expands and moves earlier in the treatment pathway in neoadjuvant and adjuvant settings, identifying patients at risk of developing irAEs is important to minimise the risk of serious toxicities while maximising the treatment benefit gained by patients.

There are currently no established predictive tools or biomarkers that can help detect patients at risk of irAEs. Clinicians mainly rely on patients’ past clinical history including presence of autoimmune disease (AD) to provide a risk estimation. However, as patients with ADs have been excluded from most clinical trials of ICIs, it is hard to determine the effectiveness of this strategy. Given the estimated overall prevalence of autoimmune diseases of 7.6–9.4%, a high proportion of patients would miss out on potentially lifesaving cancer treatment were this to be used as a screening tool^2^.

While the mechanism of irAEs is still unclear, host genetic factors are hypothesized to be key determinants. Associations between germline genetic polymorphisms and toxicity have already been established for several chemotherapeutic agents^3^. For example, variants in the dihydropyrimidine dehydrogenase *(DPYD)* gene have been shown to be associated with severe toxicities and are now screened for prior to administration of fluorouracil-based chemotherapy^3^.

**Fig. 1:**
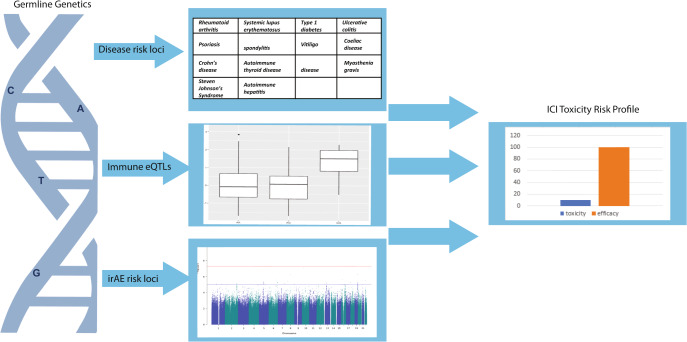
Germline genetic profiling may assist in generating patient ICI induced toxicity profiles. An ICI toxicity risk profile incorporating germline genetic factors linked with autoimmune disease risk loci, immuno-modulatory eQTLs and irAE associated loci would aid in toxicity management and decision-making to maximise treatment benefit while reducing the risk of serious irAE.

This review aims to summarise the clinical presentation of ICI-induced irAEs and the current evidence regarding germline factors and predisposition towards irAEs. As shown in Fig. 1, germline genetics may explain a significant proportion of the variation in patients’ risk of irAEs. We hypothesise that risk is conferred via associated or intermediate phenotypes such as autoimmune diseases and levels of proteins involved in the cancer immunity cycle. Novel genetic determinants will only be uncovered by large-scale studies of common and rare variants in patients treated with ICIs.

## The spectrum and challenges of immune-related adverse events

Given their similar presentation to autoimmune type events, irAEs are thought to be caused by an over-active immune response and disruption to immune homeostasis^1^. IrAEs can affect multiple organ systems including skin (manifesting as rash, pruritus, vitiligo), endocrine (thyroid, pituitary, adrenal disorders), gastrointestinal (colitis), lung (pneumonitis), musculoskeletal (arthralgia, myalgia) and liver (hepatitis). Toxicities are graded based on the Common Terminology Criteria (CTCAE) for adverse events and are usually managed using oral, intravenous high-dose steroids, or immuno-modulatory agents. Early recognition and treatment of irAE is essential for symptom resolution. Some irAEs present similarly to chemotherapy or targeted therapy induced adverse events but their underlying cause and treatment approach may differ. For example, diarrhoea induced by ICIs is likely due to immune-mediated colitis requiring treatment with steroids, whereas chemotherapy causes direct damage to the intestinal mucosa itself. Skin toxicities are known adverse events of chemotherapies, targeted agents (BRAF inhibitors and EGFR inhibitors) and ICIs. Some skin toxicity presentations are more treatment-type specific, such as hand-foot syndrome with chemotherapy and vitiligo with ICIs. Rash and pruritis are common to both chemotherapy and ICIs, but the risk of both toxicities are higher with ICI^4^. Pneumonitis, more common following treatment with anti-PD-1 than other ICI, is an example of an irAE that is likely immune related in patients on concurrent ICI and chemotherapy.

### Toxicity profile of different ICI therapies

The incidence and severity of irAEs varies according to the type of ICI therapy being used (e.g. single agent PD-1/PDL-1 inhibitors or CTLA-4 inhibitors) and whether single-agent or combination regimens are administered. Table 1 shows the frequencies of organ-specific irAEs in the context of single and combination agent ICI treatment^5–7^. Anti-PD-L1 treatment generally leads to fewer any grade or ≥grade 3 irAEs compared to anti-PD-1, possibly due to lack of inhibition of PD-L2 ligand which is involved in T cell regulation^8^. Colitis, hypophysitis and rash are more common with anti-CTLA-4 therapy, while pneumonitis, hypothyroidism, musculoskeletal toxicities and vitiligo occurred more often with anti-PD-1 therapy^5^. Higher incidences of serious ≥ grade 3 irAEs have been observed in patients treated with combination ICI regimens compared to monotherapy ICI regimens. In a phase 3 melanoma trial of 945 patients, 55% of patients receiving a doublet ICI regime of anti-CTLA-4 and anti-PD-1 experienced ≥grade 3 irAEs^9^. The incidence of irAEs following single-agent ICI treatment is lower, particularly with anti-PD-1 or anti-PD-L1 agents (~14% ≥ grade 3 events)^5,6,10^. A meta-analysis reported fatality rates related to irAEs ranged from 0.36–1.23% in patients receiving monotherapy and combination ICI treatment respectively^11^. Colitis and myocarditis were frequent causes of deaths from combination ICI therapy, whereas pneumonitis, hepatitis and neuro-toxicities most commonly contributed to anti-PD-1 or PD-L1 related fatalities^11^.

**Table 1:** Frequency of different side effects by ICI treatment type.

ICI agents	Anti-PD-1		Anti-PD-L1		Anti-CTLA-4		Anti-CTLA-4 and anti-PD-1	
irAE	Max frequency of any grade events (%)	Max frequency of ≥Grade 3 events (%)	Max frequency of any grade events (%)	Max frequency of ≥Grade 3 events (%)	Max frequency of any grade events (%)	Max frequency of ≥grade 3 events (%)	Max frequency of any grade events (%)	Max frequency of ≥Grade 3 events (%)
Pneumonitis	2.4–3.6%	0.70%	0.00%	0.00%	0.40%	0.1%	5.3–7.8%	0.2–1.1%
Cutaneous	4–28.5%	0–1.3%	<0.1–16%	0–0.6%	1.1–25.9%	0–1.3%	37.7–65%	0.3–7.6%
Diarrhoea	12.1–13%	1–1.5%	7.3–16.3%	0–0.6%	27.00%	5–7.4%	22.3–46.7%	0–15.8%
Colitis	0.7–2%	0.4–2%	0.90%	0.30%	5.7–8%	4.1–5%	46.00%	16.00%
Hepatitis	0.1–7%	0–1.5%	0.4.%	0–0.4%	0–2.6%	0–0.9%	13.4–29.5%	1.1–15.4%
Thyroiditis	1.6–9.7%	0.10%	0–2.2%	0.00%	0.5–2%	0.00%	24–27.2%	0.3–1.3%
Pituitary	0.20%	0.10%	0.00%	0.00%	1.2–4%	0.80%	1.4–4%	0.4–2.4%
Adrenal	0.00%	0.00%	0.70%	0.4%%	0.40%	0.20%	0.3–3.5%	0.2–1.4%
Diabetes	0.10%	0.10%	0.00%	0.00%	0.00%	0.00%	0.5%	0.5%
Neurological	6.00%	2.00%	<0.1%	<0.1%	3.80–4.5%	1.9%	12.00%	–
Renal	2.7%	<0.1–0.4%	0.3%	<0.1%	–	–	5.1–8.6%	0.5–1.7%
Rheumatological	0.1–43%	0–0.2%	0–6.2%	0.00%	0–2.1%%	0–0.2%	5–14%	<1–1%
Ocular	All ICI < 1%	–	–	–	–	–	–	–

Source data: Khoja et al., *Ann. Oncol.* 2017^5^, Haanen et al., *Ann. Oncol*. 2017^6^, Electronic Medicines Compendium^7^.

*ICI* immune checkpoint inhibitor, *irAE* immune-related adverse event, *Max* maximum.

### Patient and tumour characteristics that may impact risk of irAEs

Chennamadhavuni et al have reviewed the patient and tumour characteristics that are associated with risk of irAEs^12^. It is difficult to make general statements about individual risk factors as their influence is context-dependent, varying by type of ICI used and organs at risk. There is a general agreement across studies that endocrine toxicities and pneumonitis are more common in younger patients while skin toxicities are more frequent in older patients^13,14^. There are reports that sex influences risk, with thyroid irAEs being more common in women while neurological, dermal and vascular events occurred more in men^15^. Higher body mass index (BMI) and performance status have also been associated with increased risk of irAE^16,17^. Specific co-morbidities can increase the risk of certain irAEs, for example, chronic obstructive pulmonary disease is associated with an increased risk of pneumonitis^18^.

One study reported different frequencies of anti-PD-1 related irAEs in patients with different cancer types, where more gastrointestinal and skin irAEs were found in melanoma patients while pneumonitis was more common in lung and renal cancer patients^5^. When considering incidence of any grade or ≥grade 3 irAEs, Wang et al found no significant difference in incidence by cancer type, although melanoma patients did experience the highest incidence of any grade irAEs^10^.

### Time to onset of irAE

**Fig. 2:**
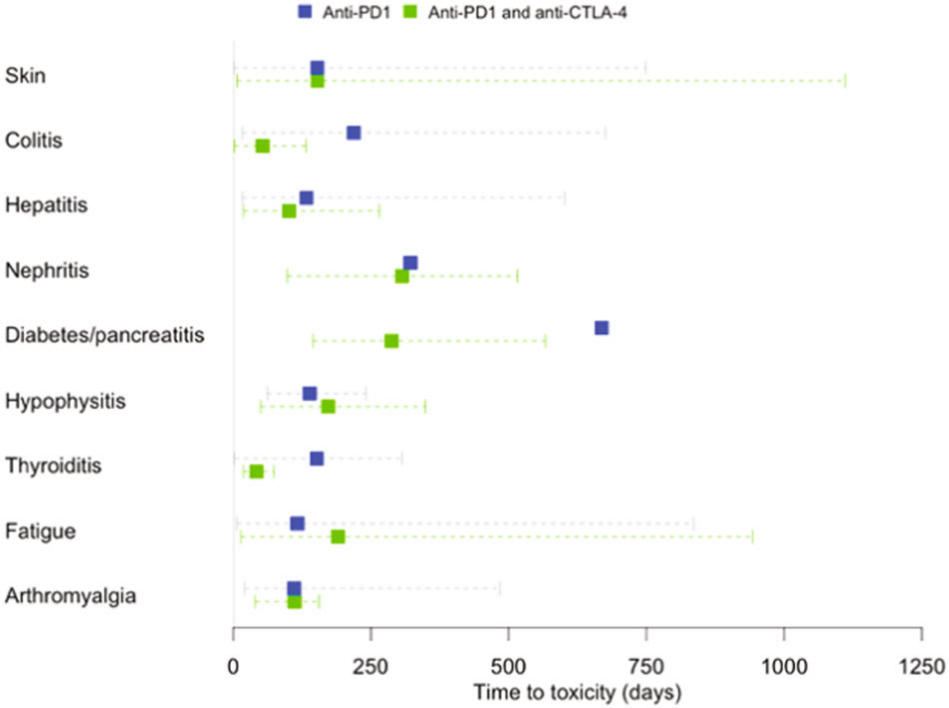
Time to onset of irAE based on the ICI GENETICS Study dataset (IRAS 237779, CPMS 39433). Time to toxicity for different irAE according to whether patients in the ICI Genetics study were treated with single anti-PD-1 or combined anti-CTLA-4 and anti-PD-1 therapy. The square box and dotted lines refer to the mean and range of the time taken from the treatment start date to development of toxicity in patients treated with anti-PD1 therapy (blue) (*N* = 92) and anti-PD1 and anti-CTLA-4 therapy (green) (*N* = 21).

The timing of irAEs vary according to the type of toxicity and ICI treatment. In an analysis of 8436 patients treated with ICI therapies, the pooled median time to onset of any toxicity of any grade ranged from 2.2 to 14.8 weeks^19^. Infusion reaction, skin and gastrointestinal events had the shortest median onset time, whereas renal toxicities occurred the latest following all ICI therapies^19^. When considering all ≥grade 3 events, the median time to onset for a serious event was 7.9 weeks for patients treated with a doublet ICI regime, 7 weeks for anti-CTLA-4 and 27.5 weeks for anti-PD-1/PDL-1 therapies^19^. An ongoing multi-centre study conducted in the UK also found that patients on combination ICI therapy tend to experience irAEs earlier than those who are on monotherapy (Fig. 2).

Some irAEs can persist and have long-term effects. The median time to resolution of irAEs can range from 0.1 to 54.3 weeks, with the longest to resolve being endocrine events^19^. In a real world analysis of 437 patients, 35.2% of irAEs reported lasted ≥6 months^20^. The lasting impact of ICIs was also demonstrated objectively in a study of patients with anti-CTLA-4 induced enterocolitis, where colitis was still evident on endoscopy for a median of 4 months after symptoms onset^21^. Other irAEs that can continue beyond treatment completion include ICI-induced type 1 diabetes and inflammatory arthritis^22,23^. These toxicities cause considerable morbidity, reliance on lifelong medication and expensive costs to healthcare systems.

ICI-induced toxicities have also been reported up to almost one year after cessation of ICI therapy^6^. Nigro et al assessed 436 patients treated with anti-PD-1/PD-L1 therapies and found that late irAEs occurring >12 months after cessation of treatment were experienced in a third of patients, with 4.8% of cases being serious events^24^. A review analysing registration trials that lead to ICI approval by the U.S Food and Drug Administration (FDA) and real-world data from melanoma patients identified that 6.9% of irAEs occur more than one year after treatment initiation^20^. Late-onset toxicities that have been reported include Raynaud’s phenomenon, which was observed >20 months after starting combination immunotherapy^25^.

Steroids, the main treatment used to counteract irAEs can cause debilitating effects on bone health, adrenal insufficiency and diabetes. Various immuno-modulatory therapies may be required to treat serious recurrent irAEs which can cause profound immunosuppression. The significant impact of irAEs on patients’ long-term health and quality of life need to be considered alongside the potentially longer survival benefit gained from ICI therapy. Predictive tests able to estimate both a patient’s likelihood of experiencing toxicity and a patient’s likelihood of a survival benefit from ICIs would be very helpful in treatment decision-making.

### ICI therapy in patients with autoimmune disease

Having concurrent AD is not uncommon in cancer patients. 13.5% of lung cancer patients in the US were found to be diagnosed with AD^26^. Studies have begun to investigate whether single-agent ICIs can be tolerated by these patient groups. Menzies et al found 38% of patients with AD treated with anti-PD-1 had an exacerbation of their AD requiring immunosuppression, but these were mainly mild events^27^. In this study, 29% of patients with AD developed an irAE and 10% experienced a grade 3 event, which is comparable to toxicity rates observed in clinical trials^27^. However, one study found 33% of patients with AD developed grade 3 irAEs on ipilimumab, indicating that patients with AD receiving anti-CTLA-4 may require more stringent monitoring^28^.

### Correlation between any grade irAE and response to ICI treatment response

There have been several reports evidencing the link between any grade ICI-induced toxicity and treatment efficacy^29,30^. A meta-analysis of 4324 patients treated with ICI found that development of all-grade irAE was correlated with a reduced risk of death (HR 0.49, 95% CI 0.38–0.62, *p* < 0.001), less risk of disease progression (HR 0.51, 95% CI 0.42–0.64, *p* < 0.001) and an odds of treatment response of 4.56 (95% CI 3.72–5.59, *p* < 0.001) compared to patients with no irAE^30^. Cancer type and drug type did not influence the results in this analysis^30^. However, a subgroup analysis found no significant correlation between grade 3/4 events or any grade events of pneumonitis with overall survival^30^. It is important to predict these more severe irAEs that are not associated with treatment benefit but instead concomitant with significant morbidity to help deliver better cancer care.

## Potential germline determinants of serious immune-related adverse events

Germline genetic factors are strong determinants of immune homeostasis and our immunological status^31^. Inherited genetic variation may influence ICI treatment outcomes, including irAE development, and explain the apparent disparity in immune responses seen between different patients receiving similar ICI agents. We highlight below genetic loci that have been found to be associated with immune traits and autoimmune diseases which warrant association testing in relation to irAEs.

### Host genetics influence levels of immuno-modulatory cells and molecules within the cancer immunity cycle

**Fig. 3:**
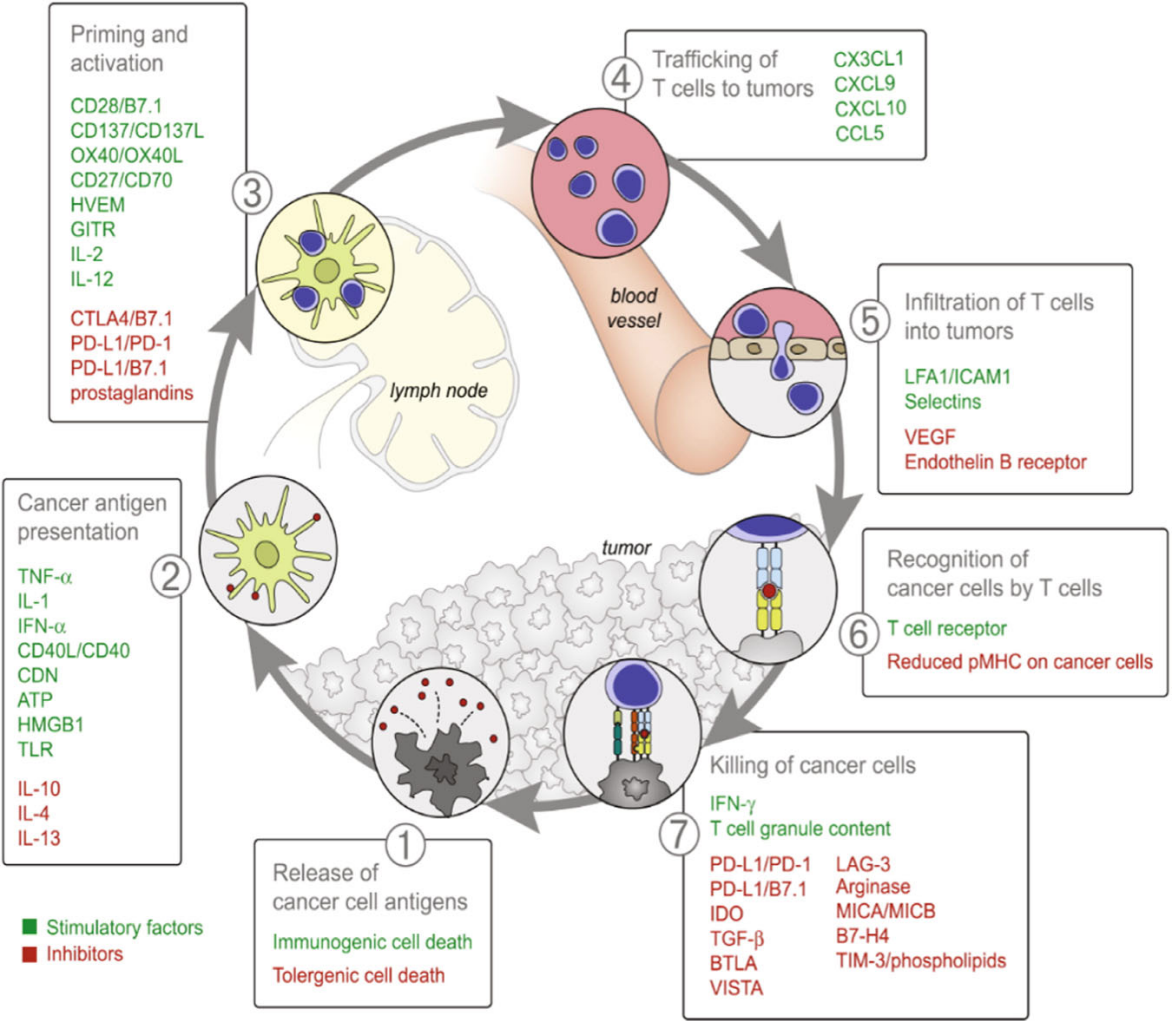
Cancer immunity cycle (Reprinted from *Immunity*, Vol. 39 Issue 1, Daniel S. Chen, Ira Mellman, Oncology meets immunology: the cancer-immunity cycle, page 10, Copyright (2013)^32^, with permission from Elsevier (Supplementary Material)). The cycle consists of step-wise events characterising the immune response against cancer cells. Various stimulatory and inhibitory immune factors play a role in each event by activating or suppressing the anti-cancer immune response. Immune checkpoints such as CTLA-4, PD-1, PD-L1 and LAG-3 act as inhibitory factors to suppress T cell activation and prevent the killing of cancer cells.

The cancer immunity cycle consists of seven step-wise events delineating the anti-cancer immune response (Fig. 3)^32^. Stimulatory and inhibitory immune factors expressed by dendritic cells, T cells and B cells regulate immune feedback mechanisms to activate the immune response while also preventing autoimmunity^32^. Host genetics explain variation in the expression and activity of various immune cell markers^31^. Many immune traits have been shown to be heritable. Those with the highest heritability include CD39 on CD4 T cells functioning as regulatory T cells and CD32 expression on dendritic cells^33^. Dendritic cells have the largest proportion of highly heritable traits followed by CD4 T cells and CD8 cells^34^. Hundreds of single nucleotide polymorphisms (SNPs) have been identified that are associated with the abundance of blood cells, immune cell marker expression and levels of molecules such as growth factors, cytokines and MHC-associated proteins^35–37^. Table 2 shows the number of genetic loci that reached GWAS significance for expression of immuno-modulatory traits from the GWAS catalogue from March to April 2021^37^.

**Table 2:** Genetic variants associated with immune-modulatory traits in the cancer immunity cycle.

Immune traits	No. of SNPs reported in the GWAS catalogue reaching GWAS significance (*P* ≤ 5 × 10^–8^) and in linkage equilibrium	Average effect sizes (beta) of alleles	Largest discovery cohort’s sample size (ethnic population)	Largest replication cohort sample size (ethnic population)	Reference
TNFα	1	2.13	13,577 (Finnish)	NA	^89^
IL-1α	1	0.74	790 (Hispanic, European, Asian, South Asian or African American)	NA	^90^
IL-1β	1	0.33	997 (European)	338 (Arab, South Asian and Filipino)	^91^
IFNα	1	1.80	410 (European)	1165 (European, African American)	^92^
CD40	2	0.89	982 (Scottish)	NA	^93,94^
CD40L	1	0.96	653 (European)	317	^95^
IL-10	8	0.08	764 (Hispanic, European, Asian, South Asian or African American)	425 (West African)	^90,96^
IL-4	1	0.40	764 (Hispanic, European, Asian, South Asian or African American)	NA	^90^
IL-13	1	0.42	3557 (Finnish)	NA	^97^
^a^CD27/CD70	2	0.64	997 (European)	338 (Arab, South Asian and Filipino)	^91^
IL-2	1	0.27	764 (Hispanic, European, Asian, South Asian or African American)	NA	^90^
^a^IL-12	5	0.21	9263 (Finnish)	NA	^94,97^
CCL5	1	0.33	3421 (Finnish)	NA	^97^
^a^LFA1/ICAM1	6	3.47	22,435 (European)	9813	^89,91,98,99^
^a^VEGF	18	0.17	13,577 (Finnish)	2800 (European, Sorbian)	^36,89,97,100,101^
IFNγ	2	0.31	7701 (Finnish)	NA	^97^
^a^MHC class I polypeptide-related sequence A (MICA)	27	0.77	997 (European)	338 (Arab, South Asian and Filipino)	^91,93^
MHC class I polypeptide-related sequence B (MICB)	8	0.63	997 (European)	338 (Arab, South Asian and Filipino)	^91^

Variants that reached GWAS significance (*P* ≤ 5 × 10^–8^) were identified from the GWAS catalogue from March to April 2021^37^. The sample sizes of the largest discovery cohort used to identify the SNP at GWAS significance are provided. Where an independent validation cohort had also been used details of the largest replication cohort are also provided. For a SNP to be included in this table, the following summary statistics had to be available: effect allele, non-effect allele, beta and *P*-value (minimal statistics required for including a variant in a polygenic risk score). Linkage disequilibrium (LD) between the lead SNP in a region and any other SNPs in the region had to be <0.8. The average absolute beta value of all SNPS is reported for traits with >1 correlated SNP. SNPs that had effect sizes reported in units measuring change of gene/protein expression were excluded.

*GWAS* genome-wide association study, *NA* not available, *SNPs* single nucleotide polymorphism.

^a^Some SNPs for this trait have beta effect sizes that are inverse transformed and/or scaled to standard deviation units.

Immune checkpoints (CTLA-4, PD-1, PD-L1) targeted by ICI therapies elicit an inhibitory effect in the priming (step 3) and cancer-cell killing (step 7) phases of the cycle^32^. *CTLA-4* gene polymorphisms have been shown to contribute to susceptibility to Graves’ disease, autoimmune hypothyroidism and type 1 diabetes^38^. The most likely causal polymorphisms, in a non-coding 3’UTR region, affect *CTLA-4* expression and are presumed to increase T-cell reactivity^38^. A missense variant in *CTLA-4*, Y60C was also found to be associated with early-onset Crohn’s disease^39^. Variants in the IL-23 and Th1 helper pathways, which are involved in immune regulation of exogeneous antigens have also been implicated in immune-mediated and autoimmune conditions^40^. These include variants at the *TYK2* locus, IL-12 and IL-12R pathways, *STAT3* and *STAT4* and *NFKB1* family^40^.

Two SNPs, rs6673928 and rs6695772, which are expression quantitative trait loci (eQTLs) influencing *IL19* (an immuno-modulatory cytokine*)* and *BATF3* (essential for CD8 + dendritic cell development) expression respectively, were shown to be significantly associated with overall survival in melanoma patients^41^. Such SNPs could potentially be used to predict prognosis for an immunogenic malignancy like melanoma.

### Genetics factors associated with autoimmune diseases

The close phenotypic similarity between patients with irAE and patients with autoimmune disease (AD) justifies further investigation into whether the resemblance is explained by shared genetic factors. Genome-wide association studies (GWAS) have identified hundreds of risk loci for over sixty autoimmune conditions^31^. Each locus is marked by one or more polymorphism each with small to moderate effect sizes. The loci tend to map to non-coding regions enriched in distant regulatory elements^42^. Table 3 shows the number of genetic loci in linkage equilibrium that reached GWAS significance for common autoimmune diseases traits from the GWAS catalogue from March to April 2021^37^.

**Table 3:** Genetic variants associated with common autoimmune disease traits.

Autoimmune disease trait	No. of SNPs reported in the GWAS catalogue reaching GWAS significance (*P* ≤ 5 × 10^–8^) and in linkage equilibrium	Average effect sizes (OR) of alleles	Largest discovery cohort’s sample size (ethnic population)	Largest replication cohort sample size (ethnic population)
Rheumatoid arthritis	186	1.16	22,628 cases, 288,664 cases (European, East Asian)	24,107 cases, 79,295 controls (East Asian, European)
Psoriasis	127	1.75	11,988 cases, 275,335 controls (European)	11,301 cases, 19,879 controls (European)
Crohn’s disease	180	1.23	19,085 cases, 34,213 controls (European)	16,619 cases, 31,766 controls (European, Iranian, Indian, East Asian)
Ulcerative colitis	80	1.32	12,924 cases, 21,442 controls (European)	25,683 cases, 17,015 controls (European)
Systemic lupus erythematosus	184	1.41	11,590 cases, 15,984 controls (European, African American, Hispanic)	1387 cases, 28,564 controls (Japanese)
Ankylosing spondylitis	55	3.74	8726 cases, 34,213 controls (European)	2111 cases, 4483 controls (European)
Autoimmune thyroid disease	141	1.48	30,234 cases, 724,172 controls (European)	7891 cases, 8351 controls (Han Chinese)
Coeliac disease	59	1.29	11,489 cases, 22,308 controls (European)	12,041 cases, 12,228 controls (European, Indian)
Type 1 diabetes	66	1.30	9934 cases, 16,956 controls (European)	4329 cases, 9543 controls (European)
Vitiligo	70	1.37	2853 cases, 37,405 controls (European)	6623 cases, 10,740 controls (East Asian)
Addison’s disease	10	2.23	1223 cases, 4097 controls (European)	NA
Myasthenia gravis	8	1.68	972 cases, 1977 controls (European)	423 cases, 467 controls (European)
Steven Johnson’s Syndrome	5	2.53	424 cases, 1881 controls (European)	102 cases, 469 controls (East Asian, Indian, Brazilian)
Autoimmune hepatitis	1	2.90	649 cases, 13,436 controls (European)	451 cases, 4103 controls (European)

Variants that reached GWAS significance (*P* ≤ 5 × 10^–8^) were identified from the GWAS catalogue from March–April 2021^37^. The sample sizes of the largest discovery cohort used to identify the SNP at GWAS significance is provided. Where an independent validation cohort had also been used, details of the largest replication cohort are also provided. For a SNP to be included in this table, the following summary statistics had to be available: effect allele, non-effect allele, OR, and *P*-value (minimal statistics required for including a variant in a polygenic risk score). LD between the lead SNP in a region and any other SNPs in the region had to be <0.8. The average absolute OR of all SNPS associated with the relevant trait is reported.

*GWAS* genome-wide association study, *OR* odds ratio, *NA* not available, *SNPs* single nucleotide polymorphism.

The genetics of AD is complicated by genetic pleiotropy, which describes a single gene or variant affecting multiple traits^31^. The A allele of rs2476601, mapping to *PTPN22* has been identified as a susceptibility allele in genome-wide studies of multiple ADs^43,44^. Other variants have been associated with multiple ADs at genome significance but with opposite effects^45^.

### HLA genes

The human leucocyte antigen (HLA) region, a 3.6 Mb region on chromosome 6 has been linked to multiple ADs^42^. Disease-linked variants at this locus often have the strongest effect sizes of all variants detected by GWAS. Variants in the genes encoding class I HLA proteins (HLA-A, HLA-B and HLA-C, expressed on all nucleated cells) have been shown to be strongly associated with ankylosing spondylitis (e.g. HLA-B27), Graves’ Disease (e.g. HLA-C*07), Type I diabetes (e.g. B*39) and multiple sclerosis (e.g. C*05)^46^. Associations between variants in class II proteins (HLA-DP, HLA-DM, HLA-DOA, HLA-DOB, HLA-DQ and HLA-DR expressed by B cells, antigen-presenting cells and activated T cells) have been identified for Graves’ disease (HLA-DR3), systemic lupus erythematosus (HLA-DR3), Hashimoto’s thyroiditis, myasthenia Gravis (HLA-DR3), Addison’s disease (HLA-DR3), rheumatoid arthritis (e.g. HLA-DRB1), coeliac disease (e.g. HLA-DR3-DQ2), multiple sclerosis (HLA-DR3) and type 1 diabetes (DR3)^40,46^. HLA variation is also known to be important in explaining a patient’s response to specific drugs; HLA-B*57:01 is screened for because of its association with severe hypersensitivity reaction to Abacavir^47^.

### Pharmacoethnicity

A large proportion of GWAS of ADs have studied European populations and while many of the identified susceptibility alleles have been replicated in Asian and African populations, some polymorphisms related to ADs such as rheumatoid arthritis have disease susceptibility effects that are variable across different ethnic groups^48^. Pharmacoethnicity needs to be taken into consideration when designing studies to identify variants associated with response to treatments like ICIs. Strong associations of HLA-B*1502 with carbamazepine-induced Steven Johnson’s Syndrome have been observed in the Han Chinese population but not in European populations^49,50^. Other pharmacogenetically relevant variants such as the *DPYD* variants recommended for testing by the clinical pharmacogenomics implementation consortium (CPIC) and Dutch Pharmacogenetics working group have lower frequencies in Asian and African populations compared to European populations^51,52^. These differences limit their utility in multi-ethnic populations and demonstrate the need for identification of relevant variants in diverse populations.

### Are genetic variants associated with response to ICIs also determinants of risk of irAEs?

Given the association between toxicity and response to ICIs discussed above, it would be interesting to determine whether SNPs associated with response are also associated with irAEs. Chat et al investigated 25 SNPs associated with three or more ADs in 436 melanoma patients and found that rs17388568 was significantly associated with favourable response to anti-PD-1 treatment and rs1893217 was significantly associated with worse outcome in patients treated with anti-CTLA-4^53^. rs17388568 maps to a locus containing *IL-2, IL-21* and *ADAD1* that have been associated with allergy, colitis and type 1 diabetes and rs1893217 maps to *PTPN2* which has a negative regulatory role in cytokine signalling and has been linked with rheumatoid arthritis^42,54^. rs2282055 and rs4143815, mapping to PD-L1 have also been shown to be associated with better treatment responses in non-small cell lung cancer (NSCLC) patients treated with nivolumab^55^. These SNPs have been associated with increased PD-L1 expression and several AD but further functional evaluation is required^55^. In a study of 166 SNPs mapping to immune-related genes in 94 NSCLC patients, Refae et al found distinct SNPs associated with response and toxicity^56^. They found that treatment response was significantly correlated with SNPs related to the tumour microenvironment, whereas SNPs associated with toxicity were predominantly target cell-related^56^. Large, well-powered studies will be required to determine whether germline variants impact on both toxicity and outcome.

## Current efforts to identify genetic factors influencing irAE development

Table 4 summarises the existing studies that have tested genetic variants for associations with irAEs and highlights variants that have been found to be significantly associated with irAE development.

**Table 4:** Genetic variants associated with irAE development.

Author	Sample size	Cancer type	Type of ICI studied	Genetic analysis approach	Toxicity type	Chr	SNPs	Effect size (95% CI)	*P* value	Mapped gene/nearest genes
Abdel-Wahab^102^	89 (44 cases, 45 controls)	Melanoma	Anti-CTLA-4, anti-PD-1/PDL1, combined ICI	All tagging SNPs	≥Grade 2 irAE	5	rs11743438	4.30 (2.3–8.0)	5.56 × 10^–6^	*GABRP*
						5	rs11743735	4.50 (2.3–8.8)	8.34 × 10^–6^	*GABRP*
						20	JHU_20.57183980	6.90 (2.7–17.6)	8.85 × 10^–6^	*DSC2*
						2	rs56328422	4.20 (2.1–8.4)	4.14 × 10^–5^	*BAZ2B*
						3	rs35807769	4.90 (2.2–11.1)	6.07 × 10^–5^	Near genes *ZPLD1* and *MIR548AB*
						5	rs3026321	19.80 (2.6–152.7)	6.31 × 10^–5^	*SEMA5A*
						5	rs1276216	6.40 (2.3–17.6)	8.18 × 10^–5^	Near genes *LOC105374704* and *CDH6*
						2	rs919682	4.60 (2.1–10.1)	8.29 × 10^–5^	*OSBPL6*
						2	JHU_2.179128562	4.60 (2.1–10.1)	8.29 × 10–5	*AGPS*
						21	rs239731	11.30 (2.5–50.4)	8.56 × 10^–5^	*LOC102724355*
						5	JHU_5.9269447	3.50 (1.9–6.5)	8.61 × 10^–5^	*SEMA5A*
						2	rs359975	8.50 (2.4–29.9)	9.41 × 10^–5^	Near genes *CFAP65* and *LOC100129175*
						3	rs11711517	0.16 (0.07–0.37)	2.39 × 10^–6^	*LOC105377125*
						15	JHU_15.93602126	0.24 (0.13–0.46)	8.89 × 10^–6^	*RGMA*
						15	JHU_15.93604000	0.24 (0.13–0.46)	8.89 × 10^–6^	*RGMA*
						18	rs470753	0.16 (0.07–0.40)	1.65 × 10^–5^	Near genes *LINC01924* and *CDH7*
						15	rs4778080	0.24 (0.12–0.47)	1.92 × 10^–5^	*RGMA*
						12	rs2117997	0.22 (0.10–0.45)	2.44 × 10^–5^	*ANKRD42*
						6	rs55733913	0.16 (0.06–0.42)	3.98 × 10^–5^	*PACRG*
						12	rs7954686	0.16 (0.06–0.42)	3.98 × 10^–5^	*FAR2*
						3	rs6440251	0.13 (0.04–0.40)	5.15 × 10^–5^	*LOC105374140*
						14	JHU_14.101799748	0.11 (0.03–0.38)	5.20 × 10^–5^	Near genes *LINC02285* and *LINC00524*
						18	rs4800887	0.25 (0.13–0.50)	5.74 × 10^–5^	Near genes *CDH2* and *MIR302F*
						3	rs162263	0.24 (0.12–0.49)	5.88 × 10^–5^	*ROBO1*
						9	rs10814859	0.24 (0.12–0.49)	5.88 × 10^–5^	*GLIS3*
						3	kgp3960064	0.28 (0.15–0.53)	6.76 × 10^–5^	*ROBO1*
						3	rs2062059	0.28 (0.15–0.53)	7.37 × 10^–5^	Near genes *DCBLD2* and *MIR548G*
						8	rs12548560	0.28 (0.14–0.53)	8.95 × 10^–5^	*PVT1*
						6	rs66502444	0.16 (0.06–0.43)	9.11 × 10^–5^	*PACRG*
						8	rs6993547	0.16 (0.06–0.43)	9.11 × 10^–5^	*PREX2*
Bins^57^	322 (96 cases, 226 controls)	NSCLC	Anti-PD-1	Small scale candidate study	Elevated liver transaminases	12	rs2301756*	2.31 (1.02–5.21)^a^	0.041	*PTPN11 (SHP2)*
					Rheumatological toxicity	12	rs2069705*	6.04 (1.53–23.86)^a^	0.019	*IFNG*
					Treatment-related adverse events	2	rs2227981*	0.45 (0.21–0.98)^a^	0.041	*PDCD1 804C*>*T (PD-1)*
Queirolo^103^	173 (10 cases, 163 control)	Melanoma	Anti-CTLA-4	Small scale candidate study	Endocrinopathies	2	rs4553808 (A/G or G/G genotypes)	0.09 (0.02–0.43)^a,b^	0.003	*CTLA-4–1661A*>*G*
Cappelli^59^	753 (27 cases, 726 controls)	Mixed (largest cohort Melanoma)	Anti-PD-1/CTLA-4, anti-PD-1/PD-L1+/− anti-CD73 or anti-LAG3	HLA specific approach	Inflammatory arthritis	6	HLA DRB1*04: 05	8.60 (1.70–43.40)	0.040	*HLA*
Magis^60^	163 (5 cases, 158 controls)	Melanoma	Anti-PD-1	HLA specific approach	Acute type 1 diabetes	6	HLA DRB1*04:01, DRB1*04:05, DRB1*03:01	NA	NA	*HLA*
Refae^56^	94 (15 cases, 79 controls)	Advanced cancer (largest cohort NSCLC)	Anti-PD-1/PDL1	Medium scale candidate study	Serious ≥grade 3 irAE	12	rs246079	0.09 (0.02–0.36)^c^	<0.001	*UNG*
						9	rs10964859	6.08 (1.52–24.33)^b^	0.014	*IFNW1*
						9	rs4143815	8.11 (2.09–31.40)^b^	0.003	*PD-L1*
						19	rs12979860	3.72 (1.09–12.68)^b^	0.036	*IFNL4*
						2	rs3087243	3.19 (1.01–10.27)^b^	0.048	*CTLA4*
						2	rs11571302	4.06 (1.23–13.38)^b^	0.018	*CTLA4*
						2	rs7565213	3.73 (1.14–12.19)^b^	0.026	*CTLA4*
Iafolla^62^	101 (23 cases, 78 controls)	Mixed (largest cohort triple negative breast cancer)	Anti-PD-1	HLA specific approach	≥Grade 2 irAE	6	HLA-A	1.03 (0.23–4.62)^d^	0.970	*HLA*
						6	HLA-B	1.32 (0.24–7.26)^d^	0.750	*HLA*
						6	HLA-C	0.81 (0.24–2.78)^d^	0.740	*HLA*
						6	HLA-A, -B and -C	0.58 (0.21–1.62)^d^	0.300	*HLA*
Hasan Ali^58^	102 (59 cases, 43 controls)	Advanced cancer	Anti-PD-1	HLA specific approach	Pruritus	6	HLA-DRB1*11:01	4.53 (X^2^_1,95_ = 9.45)	0.002	*HLA*
					Colitis	6	HLA-DQB1*03:01	3.94 (X^2^_1,95_ = 5.67)	0.017	*HLA*
Luo^69^	729 (95 cases, 634 controls)	NSCLC	Anti-PD-1 or combined anti-PD-1 and CTLA-4	All tagging SNPs	Thyroid disorders	6	rs9268543	NA	7.5 × 10^–7^	*HLA*
Kirchhoff^104^	69 (30 cases, 39 controls	Melanoma	Anti-CTLA-4	Whole exome sequencing and detection of ~1000 autoimmune variants	Serious ≥grade 3 irAE	19	rs504963	2.57	0.006	3’UTR of *FUT2*
						6	rs1738074	2.21	0.025	*TAGAP*
Montaudié^70^	57 (33 cases, 24 controls)	Melanoma	Anti-PD-1, Anti-CTLA-4, combined anti-PD-1 and anti-CTLA-4	All tagging SNPs	Any grade irAE	2	rs782637	NA	4.10 × 10^–8^	*PNPT1*
						2	rs78259	NA	3.40 × 10^–7^	*PNPT1*
						2	rs7882572	NA	3.80 × 10^–7^	*PNPT1*
						2	rs3762513	NA	6.60 × 10^–7^	*CFAP36*
						11	rs7481951	NA	3.10 × 10^–5^	*ANO5*
						2	rs4988956	NA	3.80 × 10^–4^	*IL1RL1*
Yano^61^	11 cases	Melanoma, NSCLC, gastric	Anti-PD-1, anti-CTLA-4	HLA specific approach	Pituitary disorders	6	HLA-DR15	NA	1.40 × 10^–3^	*HLA*
						6	HLA-DR1502	NA	0.002	*HLA*
						6	HLA-Cw12	NA	0.001	*HLA*
						6	HLA-B52	NA	0.003	*HLA*
Marschner^105^	167 (39 cases. 128 controls)	Melanoma, lung	Anti-PD-1/PDL1	Small scale candidate study	Serious ≥grade 3 irAE	5	rs2910164 (C/C genotype)	6.78 (1.87–24.60)	0.004	*MIR146A*
Groha^65^	1751 (339 cases, 1412 controls)	Mixed (largest cohort NSCLC)	Anti-PD-1/PD-L1, combined ICI, anti-CTLA-4	All tagging SNPs	All grade irAE	8	rs16906115	HR = 2.0 (1.6–2.5)	3.8 × 10^–9^	*IL-7*
						1	rs75824728*	HR = 1.9 (1.5–2.4)	8.4 × 10^–9^	*IL22RA1*
						4	rs113861051*	HR = 2.0 (1.6–2.6)	1.1 × 10^–8^	Near genes *LINC02484* and *ARAP2*
Weidhaas^68^	161 (45 cases, 116 controls)	Mixed (largest cohort melanoma)	Anti-PD-1/PD-L1	Candidate study (50 common variants mapping to regions expected to disrupt miRNAs)	≥Grade 2 irAE	7	rs9374	AUC 0.82 (in a panel)	<0.001	*RAC1*
Udagawa^66^	622 (520 cases, 102 controls)	Mixed (largest cohort lung)	Anti-PD-1	All tagging SNPs	All ≥grade 1 irAE	21	rs469490	5.15 (2.39–13.22)	2.97 × 10^–7^	Near genes *APP* and *CYYR1-AS1*
					≥Grade 1 Thyroid irAE	15	rs8023690	4.09 (2.21–8.15)	2.59 × 10^–7^	Near genes *RGMA* and *LINC02207*

Studies investigating on genetic determinants of irAE were reviewed up till October 2022. Effect sizes for all studies are reported in odds ratios and 95% CI unless indicated otherwise.

*Chr* chromosome, *CI* confidence interval, *HR* hazard ratio, *ICI* immune checkpoint inhibitor, *irAE* immune-related adverse events, *NA* not available, *NSCLC* non-small cell lung cancer, *SNPs* single nucleotide polymorphism.

^a^Univariate analysis.

^b^Under recessive model.

^c^Under dominant model.

^d^Summary statistics based on HLA-1 loci heterozygosity relative to homozygosity.

*Did not validate in independent sample sets.

Majority of the existing studies involved sample sizes of <200 patients and analysed variants in candidate genes or regions associated with immune response autoimmunity and response to systemic stress. The small sample sizes studied to date limit the chance of identifying true associations and indeed one of the larger of the existing studies, involving 322 NSCLC patients on nivolumab was unable to validate associations between lower odds of any grade toxicity in patients homozygous for *PDCD1* 804C>T (rs2227981) and higher odds of rheumatological toxicity in patients with one or more copy of *IFNG* −1616T>C in a validation cohort^57^.

As shown in Table 4, the association between HLA alleles and irAE occurrence has been explored. HLA variation tends to be associated with organ-specific autoimmune toxicities as opposed to a combined measure of irAE occurrence. HLA alleles have been shown to be associated with ICI-induced pruritus (HLA-DRB1*11:01, OR 4.53, *p* = 0.002) colitis (HLA-DQB1*03:01, OR 3.94, *p* = 0.017)^58^, arthritis (HLA-DRB1*04:05, OR 8.6, *p* = 0.04)^59^, type 1 diabetes (HLA-DRB1*03 and HLA-DRB1*04 haplotypes)^60^ and pituitary irAE (81.8% HLA-DR15 vs 33.5% in healthy controls, *p* = 0.0014)^61^. In contrast, analysis of cases of severe irAEs such as fulminant type 1 diabetes have failed to identify any association between HLA alleles and irAEs^62–64^. Validating the hypothesis that germline variants associated with AD or immunomodulation may be important in explaining risk of irAEs; three of the variants identified as associated with irAE (Table 4) were associated with ADs (Table 3); rs1738074 (coeliac disease risk), HLA-DQB1*03:01 (systemic lupus erythematosus risk) and rs3087243 (rheumatoid arthritis risk).

The first GWAS of irAE was posted on MedRXiv in April 2022^65^. This study of 1751 patients on ICIs identified a genome-wide significant association between a SNP mapping to *IL7*, rs16906115, and any grade irAE toxicities^65^. rs16906115 replicates in two independent cohorts and is the first genetic variant associated with irAEs to have been identified using large sample sizes and validation cohorts^65^. The vast majority of patients included in this study were on PD1/PD-L1 inhibitors as single agents^65^. It remains to be determined if rs16906115 is also associated with CTLA-4 driven irAEs. A second GWAS of any grade irAE toxicity has also recently been published^66^. The study identified 27 SNPs associated with any grade nivolumab induced irAE with a *P* < 1 × 10^–4^ including rs469490, which lies upstream to *APP* which has been linked to Crohn’s disease^67^. A subgroup analysis identified rs8023690 as a potential predictive marker for hypothyroidism (OR 4.09, 95% CI 2.21–8.15, *P* = 2.58 × 10^–7^)^66^. Majority of patients included in the study experienced an irAE; only 86 and 16 controls were included in the discovery and replication phases, respectively^66^. The study was only powered (at 80%) to detect large effect sizes of common SNPs (minor allele frequency >0.1 and OR > 2.2).

Weidhaas et al tested a panel of germline variants predicted to disrupt miRNA binding in 62 melanoma patients and 99 patients with other cancer types including prostate cancer treated with anti-PD-1/PD-L1. The 50 variant panel, applied using four different classifiers achieved an AUC of ~0.80 for the prediction of ≥grade 2 irAEs in their training and validation cohorts^68^. One of the markers included in the classifier rs9374 was associated with a nine-fold increased risk of ≥grade 2 irAEs. It would be interesting to examine this marker in larger datasets. A polygenic risk score for hypothyroidism developed using UK Biobank data consisting of 1502 SNPs was found to predict thyroid irAE in NSCLC patients treated with ICIs^69^. This finding suggests the potential utility of applying risk scores to generate irAE risk profiles. Exome sequencing studies of patients treated with ICIs have also only been performed in small cohorts of patients. Montaudié et al performed exome sequencing of 57 melanoma patients with 57 patients also available for validation^70^. The authors concluded that germline variants had limited impact on irAE occurrence, however much larger cohorts are required to test this^70^.

## Future directions

55% of patients treated with combination ICIs and 14–33% of patients treated with single-agent ICIs experience one or more ≥grade 3 irAEs across their treatment course. We have highlighted above the rationale for utilising germline testing to predict those at risk of developing these serious toxicities. As mentioned, any grade toxicity has also been associated with improved outcome and so the risk of toxicity will need to be balanced with chance of efficacy to optimise cancer care. Thus far the majority of studies testing individual genetic factors for associations with irAEs have been limited by their sample size and lack of independent validation of their findings. Large cohort studies with selection of an appropriate methodology are vital to produce meaningful associations that can be translated to clinical practice.

### Methods of identifying genetic variants associated with irAE

One established hypothesis-free method of determining potential genetic determinants of human traits is GWAS^71^. GWAS have contributed towards the understanding of disease susceptibility, biomarker discovery and personalised therapeutic options^72^. Most genetic studies of AD have implemented GWAS to identify common predisposing variants of specific AD. This approach can similarly be applied to identify common variants predisposing to irAE risk using either SNP array technology or low pass (0.5–1x) whole genome sequencing^73^. Standard depth (~30x) whole genome sequencing (WGS) is a more comprehensive genotyping modality and allows rare variants to be accurately assessed. It is however a considerably higher cost approach compared to SNP array genotyping or low-pass sequencing both in terms of experimental cost and analytical complexity^74^. While whole exome sequencing is more cost-effective than WGS, it does not allow assessment of non-coding regulatory regions that could be functionally important^74^.

GWAS do not normally identify causal variants or target genes, these are identified by fine mapping variants in linkage disequilibrium with the SNPs associated with the phenotype of interest to identify the variants that should be studied further in functional analyses^72^. GWAS have also identified novel pathways and mechanisms important in conferring phenotypes and disease risk. While GWAS do require large sample sizes, we note that pharmacogenetics studies have reported larger per allele effect sizes than have been detected in studies of complex diseases^75^. Studies of >500 patients with irAEs will therefore be powered to pick up large effect sizes in excess of an odds ratio of 2 (assuming a grade 3 toxicity incidence of ~10%). Larger studies will be required to detect the full spectrum of variants explaining an individual’s risk of developing irAEs. Encouraging results from the first GWAS with a sample size of over 1000 patients suggest that there are likely to be further germline genetic variants influencing the occurrence of irAEs^65^. As the incidence of irAE may be influenced by various treatment, disease and patient-related factors, covariates including type of ICI regime, cancer type, age, gender, BMI and performance status may need to be considered in these studies^12^.

Close consideration of the definition of cases in GWAS of irAE is also important. Identifying variants predictive of serious ≥grade 3 irAE would likely have more beneficial translational value in practice compared to predicting any grade events, although large sample sizes would be required to avoid being underpowered.

### Polygenic risk score analysis

A useful adjunct to GWAS is polygenic risk profiling using polygenic risk scores (PRS), which measure an individual’s genetic susceptibility to a disease determined by the sum of the risk alleles (weighted by the effect size of each variant) they carry^76^. As mentioned above a PRS for hypothyroidism was able to significantly predict thyroid irAE in 729 NSCLC patients (HR per SD 1.34, 95% CI 1.08–1.66, *P* = 9.73 × 10^–3^, AUROC = 0.6)^69^. In addition, Khan et al identified an association between the development of skin irAE with PRS for psoriasis in bladder cancer patients receiving anti-PD-L1^77^. Existing risk scores for relevant autoimmune diseases may have power in predicting patients at high risk of specific toxicities. Risk scores however are only going to be powerful if they explain a large proportion of the genetic risk of developing a toxicity. If only a few genetic causes of a trait are known about, the PRS has limited value. PRS would likely need to be used alongside non-genetic risk factors that are also associated with the risk of developing irAEs.

### Developing a predictive model of irAE for use in clinical practice

The challenge of identifying patients at serious risk of irAE and the potentially harmful short and long-term adverse effects warrant further investigation onto the development of a predictive model of irAE. The potential treatment benefit also needs to be considered in order to not deprive patients of an effective cancer treatment. Balance between treatment efficacy and toxicity will need to be achieved by developing predictive models to estimate both treatment outcomes to help with risk stratification.

Research findings on host genetics should be complemented with those from other ‘omics’ such as the microbiome, immunome, metabolome and the tumour microenvironment. Numerous studies have detected increased levels of cytokines and chemokines including IL-1a, IL-2, IFNα2, IL-6, IL-17 and post-treatment CXCL9 and CXCL10 as probable predictors of irAE occurrence^78–81^. Early diversification of T cell repertoire with decline in T cell clonality, increase in Th17 cells or early B cell changes have all been found to be associated with early development of irAE^82–85^. Deficiency in certain regulatory B cell phenotypes was found in patients who developed serious irAE^86^. Chaput et al showed that the composition of gut microbiota with *Faecalibacterium* and other *Firmicutes* has been associated with ipilimumab-induced colitis, whereas, the presence of *Bacteroidetes* was protective against colitis^87^. Tahir et al also found a correlation with autoantibodies anti-GNAL and anti-ITM2B with hypophysitis and anti-CD-74 with pneumonitis induced by ICI^88^.

An effective predictive model of toxicity can guide clinical decisions on personalised treatment options and safety monitoring of toxicities based on the patient’s risk of irAE. Earlier detection of patients at high risk of a particular toxicity with allow for stricter monitoring and earlier intervention to reduce the risk.

## Conclusion

A large number of genetic variants have been identified which explain variation in immune modulation and risk of AD development. These variants may also be genetic determinants of irAEs. Large genome-wide studies of thousands of patients on ICIs are needed to detect common and rare variants with clinically relevant effect sizes that can contribute towards the development of a candidate gene panel in a predictive risk model of toxicity.

### Reporting summary

Further information on research design is available in the Nature Portfolio Reporting Summary linked to this article.

## Supplementary information

Reporting Summary Checklist

## Data Availability

The datasets analysed during the current study are available in the NHGRI-EBI GWAS Catalog repository [https://www.ebi.ac.uk/gwas/].
